# Curcumin exerts a protective effect against premature ovarian failure in mice

**DOI:** 10.1530/JME-17-0214

**Published:** 2018-02-07

**Authors:** Zhengjie Yan, Youjin Dai, Heling Fu, Yuan Zheng, Dan Bao, Yuan Yin, Qin Chen, Xiaowei Nie, Qingting Hao, Daorong Hou, Yugui Cui

**Affiliations:** 1 College of Animal Science and TechnologyYangzhou University, Yangzhou, People’s Republic of China; 2 State Key Laboratory of Reproductive MedicineCenter of Clinical Reproductive Medicine, The First Affiliated Hospital of Nanjing Medical University, Nanjing, People’s Republic of China; 3 Key Laboratory of the Model Animal ResearchAnimal Core Facility of Nanjing Medical University, Nanjing Medical University, Nanjing, People’s Republic of China; 4 Department of Reproductive MedicineAffiliated Hospital of Nanjing University of Traditional Chinese Medicine, Nanjing, China

**Keywords:** premature ovarian failure, d-galactose, curcumin, ovary aging, oxidative stress

## Abstract

This study was designed to investigate the protective effect of curcumin against d-galactose (d-gal)-induced premature ovarian failure (POF) in mice. A mouse POF model was induced by subcutaneous injection of d-gal (200 mg/kg/day) daily for 42 days. Mice in the curcumin group received both d-gal treatment and intraperitoneal injection of curcumin (100 mg/kg/day) for 42 days. Ovarian function, oxidative stress and apoptosis were evaluated. The P, E2 and SOD levels were higher, and the FSH, LH and MDA levels were significantly lower in the curcumin group than those in the d-gal group. The proportion of primordial follicles was also significantly higher in the curcumin group than that in the d-gal group. In addition, curcumin treatment after d-gal administration resulted in significantly lower *Sod2*, *Cat*, 8-OhdG, 4-HNE, NTY and senescence-associated protein P16 expression levels, higher *Amh* expression levels and less apoptosis in granulosa cells than was observed in the d-gal group. Moreover, the p-Akt, Nrf2 and HO-1 protein expression levels were significantly higher and the apoptosis-related cleaved caspase-3 and -9 protein expression levels were markedly lower in the curcumin group than in the d-gal group. In conclusion, curcumin effectively inhibited d-gal-induced oxidative stress, apoptosis and ovarian injury via a mechanism involving the Nrf2/HO-1 and PI3K/Akt signaling pathways, suggesting that curcumin is a potential protective agent against POF.

## Introduction

Premature ovarian failure (POF), also called premature ovarian insufficiency (POI), affects approximately 1% of women in the general population, in whom it causes amenorrhea and hypergonadotropic hypoestrogenism before the age of 40 years (Kaufman *et al*. 1988, Waggoner *et al*. 1990, Guerrero *et al*. 2000, Bandyopadhyay *et al*. 2003). However, the pathological mechanism underlying POF remains unclear. The associated ovarian pathology is related to the toxic effects of galactose and its metabolites at both the ovarian and extraovarian levels (Campbell *et al*. 2010*a*,*b*, Rubio-Gozalbo *et al*. 2010). Moreover, there is no effective etiological treatment for POF. Hormone replacement therapy (HRT) is available to treat the symptoms of POF, and follicle donation is available for some POF patients seeking to become pregnant. However, HRT has been confirmed to confer a high risk of coronary heart disease, endometrial cancer and breast cancer in women with POF (Deady 2004).

POF is a common clinical feature of galactosemia (Waggoner *et al*. 1990, Guerrero *et al*. 2000), and women with galactosemia eventually develop POF (Kaufman *et al*. 1988). The estrous cycle of female mice is similar to but shorter than that of humans. The mouse d-galactose (d-gal)-induced POF model is used as a model of aging in mice and has been widely used to study the mechanisms underlying ovarian aging, because the accelerated aging observed in this model is very similar to observations in humans (Song *et al*. 1999, Semba *et al*. 2010). Aging is associated with increased deposition of advanced glycation end products (AGEs) and reactive oxygen species (ROS) in the myocardium, brain, liver, eye, red blood cells, kidney, bone, ovary, muscles and tendons (Kimura *et al*. 1996, Nerlich & Schleicher 1999, Schinzel *et al*. 2001, Simm *et al*. 2004, Odetti *et al*. 2005, Haus *et al*. 2007, Kumar *et al*. 2007, Hyogo & Yamagishi 2008). Many studies have indicated that AGEs and ROS exacerbate and accelerate the aging process and contribute to the early phases of age-related diseases, including atherosclerosis, cataracts, neurodegenerative diseases, renal failure, arthritis, ovarian senescence and age-related macular degeneration (Tian *et al*. 2005, Semba *et al*. 2010). d-gal administration can cause excessive ROS formation and AGE accumulation (Song *et al*. 1999, Mao *et al*. 2010, Lin *et al*. 2012). ROS-induced damage and AGE accumulation are widely accepted causes of aging that gradually damage ovarian functions (Semba *et al*. 2010). Therefore, d-gal has been used to induce the POF model (Bandyopadhyay *et al*. 2003). Curcumin (chemical name: 1, 7-bis (4-hydroxy-3-methoxyphenyl)-1, 6-heptadiene-3, 5-dione) (Aggarwal *et al*. 2005, Ak & Gülçin 2008) is an active component of turmeric rhizomes (*Curcuma longa Linn*), which contain 3–5% curcumin (Zhang *et al*. 2013). Curcumin has been scientifically demonstrated to function as an antioxidant (Fujisawa *et al*. 2004, Calabrese *et al*. 2008, Dinkova-Kostova & Talalay 2008, Augustyniak *et al*. 2010), anti-inflammatory (Aggarwal & Harikumar 2009, Wang *et al*. 2012, Ueki *et al*. 2013), anti-apoptotic (Geng *et al*. 2017) and antibacterial (Mun *et al*. 2013) substance. An increasing number of studies have shown that curcumin directly suppresses proliferation and promotes apoptosis in ovarian cancer cells (Shehzad *et al*. 2010, Watson *et al*. 2010, Terlikowska *et al*. 2014, Vallianou *et al*. 2015, Seo *et al*. 2016) and prevents the adverse effects of ovarian insufficiency (Tiwari-Pandey & Ram Sairam 2009, Voznesens’ka *et al*. 2010, Alekseyeva *et al*. 2011, Aktas *et al*. 2012), ionizing radiation (Aktas *et al*. 2012), ischemia (Eser *et al*. 2015), oxidative stress (Qin *et al*. 2015) and mycotoxins (Qin *et al*. 2015) on ovarian function. Some studies have also demonstrated that curcumin and its analogues exert a stimulatory effect on ovarian functions, because they promote proliferation and reduce apoptosis in murine ovarian cells (Voznesens’ka *et al*. 2010, Aktas *et al*. 2012) while supporting murine ovarian folliculogenesis (Voznesens’ka *et al*. 2010, Alekseyeva *et al*. 2011) and steroidogenesis (Tiwari-Pandey & Ram Sairam 2009). However, the effects of curcumin on experimentally d-gal-induced ovarian aging have not been reported. In this study, we investigate the protective effect of curcumin on ovarian aging in a d-gal-induced mouse model of POF.

## Materials and methods

### Animals and treatment

A total of 60 C57BL/6 female mice aged 7–8 weeks were used. The animals were obtained from the Animal Core Facility of Nanjing Medical University, Nanjing, China and housed under a 12-h darkness/light cycle in an animal facility with a controlled temperature (20–25°C) and humidity (40%–70%). Food and water were provided *ad libitum* throughout the study. The mice were allowed to acclimatize for 1 week. Then, they were randomly divided into the three following groups with 20 mice per group: control group, d-gal group and curcumin group. The mice in the d-gal group were subcutaneously (s.c.) injected daily with d-gal (200 mg/kg/day) for 42 days (Park & Choi 2012, He *et al*. 2017), the mice in the control group received an equal volume of saline (s.c. daily) for 42 days and the mice in the curcumin group received curcumin (100 mg/kg/day) (Chainani-Wu 2003, Aktas *et al*. 2012) via intraperitoneal injection following a daily d-gal injection for 42 days. All experiments involving animals were approved by the Institutional Animal Care and Use Committee (IACUC) of Nanjing Medical University, and the methods were conducted in accordance with the approved guidelines. On the last day of drug administration (day 42), all mice were killed while under general anesthesia (induced using an intraperitoneal injection of pentobarbital sodium (150 mg/kg)). Blood was collected through a heart puncture, the left ovary was immediately excised and stored at −80°C for biochemical analysis and the right ovary was fixed in 4% paraformaldehyde for histological studies.

### Primordial follicle counting

The right ovaries were fixed for 12 h in 4% paraformaldehyde and then embedded in paraffin. The tissues were serially sectioned (6-µm thick), mounted on glass slides and stained with H&E. The ovarian follicles were counted according to the methods described in a previous study (Tilly 2003, Bernal *et al*. 2010). Briefly, every fifth section was observed under a microscope. To avoid repeated counting of the same follicle, only follicles with a visible oocyte nucleus were included. The numbers of primordial follicles in all serial sections of an ovary were counted. The following follicle classification was used (Hirshfield & Midgley 1978, Wang *et al*. 1991, Borgeest *et al*. 2002): type 1: primordial follicle, one layer of flattened granulosa cells surrounding the oocyte; type 2: primary follicle, one to two complete layers of cuboidal granulosa cells; type 3: secondary follicle, an oocyte surrounded by more than one layer of cuboidal granulosa cells with no visible antrum; type 4: antral follicle, an oocyte surrounded by multiple layers of cuboidal granulosa cells and containing one or more antral spaces, possibly with a cumulus oophorus and thecal layer and type 5: atretic follicle, a follicle that enters a degenerative process without ovulation. The oocyte nuclei in the atretic follicles shrink, the chromosomes and cytoplasm dissolve, the granulosa layer decreases and the follicular membrane cells are hypertrophic (Paulose *et al.* 2012). The primordial follicle ratio refers to the percentage of the primordial follicle number out of the total follicles. The atretic follicles were included in the denominator when calculating the proportion of primordial follicles or total follicles.

### Sample preparation and biochemical assays

All blood samples were collected while the mice were in diestrus and allowed to clot at room temperature. Then, the samples were centrifuged at 900 ***g*** for 10 min to harvest serum. Serum biochemical parameters, including the serum follicle-stimulating hormone (FSH), luteinizing hormone (LH), progesterone (P) and estradiol (E2) levels, were measured spectrophotometrically (Eon, BioTeK, Vermont, UT, USA) using the following commercially available ELISA kits: FSH (KA2330), LH (KA2332) (Novus Biologicals, Littleton,USA), E2 (582251) and P (582601) (Cayman Chemicals, Ann Arbor, MI, USA). The ovaries were washed in ice-cold saline and homogenized in 0.1 M Tris–HCl buffer (pH 7.4). The homogenates were centrifuged at 10,000 ***g*** for 15 min, and the supernatants were centrifuged at 100,000 ***g*** for 1 h. The resulting supernatant (cytosolic fraction) was used to determine the enzymatic activity and lipid peroxidation levels. The biochemical parameters of the ovaries, including the total superoxide dismutase (SOD) activity and malondialdehyde (MDA) level, were measured spectrophotometrically using commercially available kits for SOD (A001-1) and MDA (A003-1) (Jiancheng Bioengineering Institute, Nanjing, China).

### Q-PCR

Total mRNA was extracted from the ovarian samples using the TRIzol reagent (B5704-1, Takara) according to the manufacturer’s instructions and then treated with DNase I (2212, Takara) according to the manufacturer’s protocol. The quality and quantity of the RNA were determined using a spectrophotometer (NanoDrop 2000c, Thermo Scientific). cDNA was immediately synthesized using the PrimeScript RT Reagent Kit (RR037A, Takara) according to the manual supplied by the manufacturer. Q-PCR was performed using a Light Cycler PCR QC Kit (Roche) and a 7300 Real-Time PCR System (LC96, Roche). The PCR primers are listed in Supplementary Table 1 (see section on supplementary data given at the end of this article). The housekeeping gene GAPDH was used as the internal reference. Expression of the target gene was normalized to GAPDH and calculated using the comparative quantification method (2^−ΔΔCt^). Expression of the target genes was corrected to GAPDH prior to normalization. Firstly, the Ct value of each group was subtracted from the Ct value of the internal reference gene, which was named ΔCt, as follows: ΔCt = Ct (target gene) − Ct (internal reference gene). Secondly, the ΔCt of the experimental group was subtracted from the control group, and then the inverse of all of the results was taken to obtain −ΔΔCt. Finally, the power operation of −ΔΔCt was performed in 2. The GraphPad Prism 5 software was used for chart production.

### Immunohistochemical staining

For the immunohistochemical analysis, paraffin-embedded sections were dewaxed and then subjected to heat-mediated antigen retrieval, which was performed by microwaving the sections for 20 min in 10 mM sodium citrate (pH 6.0). The sections were allowed to cool for 15 min, briefly washed in deionized water and then rinsed twice in PBS. The sections were incubated for 30 min in 5% goat serum in DPBS containing 0.1% Tween and 0.5% BSA. The sections were incubated overnight at 4°C with primary antibodies against 8-hydroxyguanosine (ab26842), 4-hydroxynonenal (ab48506), anti-CDKN2A/p16 (ab189034) (Abcam Biotechnology) and nitrotyrosine (sc-71007) (Millipore Biotechnology) at the appropriate dilutions. The secondary antibody in the Dako REAL EnVisio Detection System (K5007) (DAKO) was used to detect labeling. Then, the specimens were counterstained with hematoxylin for 1 min. All sections were incubated under the same conditions and at the same time using the same antibody concentrations. The tissue sections were observed and photographed with a microscope and semi-quantified using the Image Pro Plus 6.0 software. The integrated optical density (IOD) was collected for each photograph. Five fields for each slice (five slides per animal) were randomly selected for blinded measurements (*n* = 8 per group). The images were quantified by the immunoreactive area (IA) in μm^2^ and the IOD. The staining intensity (SI) for each image was calculated as SI = IOD/IA, and the mean with standard deviation was obtained for each series.

### *In situ* TUNEL fluorescence staining assay

The terminal deoxynucleotidyl transferase (TdT)-mediated deoxyuridine triphosphate (dUTP) nick end-labeling (TUNEL) assay was performed according to the manufacturer’s instructions (11684817910, Roche). Ovarian tissues were fixed in 4% paraformaldehyde overnight, dehydrated, embedded in paraffin, cut into 4-μm-thick sections and placed on numbered polylysine-coated glass slides. Deparafﬁnized tissue sections were incubated with proteinase K (20 mg/mL) in a humidified chamber for 15 min, and endogenous peroxidase activity was blocked by treating the sections with 3% H_2_O_2_ for 10 min. The sections were incubated with TdT labeling buffer at 37°C for 1 h in a moist chamber. Then, the sections were counterstained with DAPI. The TUNEL-positive cells stained green, and the nuclei were stained with DAPI (blue). To eliminate the histological differences between ovarian tissues, five random fields per slide (five slides per animal, eight animals per group, *n* = 8) were examined. In total, 200 random fields (5 × 5 × 8 = 200) per group were checked. The TUNEL-positive granulosa cells and the total granulosa cells in the antral follicles were counted. The rate of TUNEL-positive granulosa cells (%) in the antral follicles was analyzed using the Image Pro Plus 6.0 software.

### Western blotting

The Western blotting analysis was performed according to the methods described by Banerjee *et al*. (2012). Briefly, 50 µg of total lysate obtained from ovarian tissue was subjected to 15% polyacrylamide gel electrophoresis and then transferred to a cellulose acetate membrane. The membranes were blocked with 1× casein solution for approximately 4 h and then incubated with rabbit monoclonal anti-Akt (4685), anti-p-Akt (Ser473) (4058), anti-cleaved caspase-3 (Asp175) (9664) or anti-cleaved caspase-9 (Asp330) (7237) antibodies obtained from Cell Signaling Technology, rabbit polyclonal anti-Nrf2 (sc-722) or anti-GAPDH (H-12) (sc-166574) antibodies or a mouse monoclonal anti-HO-1 (sc-390991) antibody obtained from Santa Cruz Biotechnology in blocking buffer for 2 h at room temperature. The membranes were washed 3 times with TBST and then incubated with a goat anti-rabbit IgG HRP-conjugated secondary antibody (sc-2004, Santa Cruz Biotechnology) or a goat anti-mouse IgG HRP-conjugated secondary antibody (sc-2005, Santa Cruz Biotechnology). Then, the membranes were washed 3 times in TBST, and the blots were imaged using the ChemiDoc XRS+ Molecular Imager (Bio-Rad) with the Pierce ECL Western Blotting Substrate (32209, Thermo Scientific) and analyzed using image analysis software (ImageJ 1.42). The housekeeping protein GAPDH was used as the internal reference. The Western blotting quantification was corrected to GAPDH expression prior to normalization.

### Statistical analysis

All statistical analyses were performed using SPSS v.16. All results are shown as the mean ± standard deviation (M ± s.d.). All statistical comparisons were performed using one-way ANOVA followed by Duncan’s multiple range *post hoc* analysis. A *P* value <0.05 was considered significant.

## Results

### Protective effects of curcumin on the HPG axis and ovarian *Amh* mRNA expression

**Figure 1:**
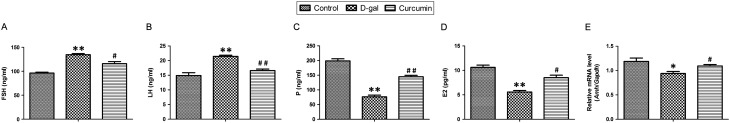
Protective effects of curcumin on the HPG axis and ovarian AMH. The serum FSH (A), LH (B), E2 (C) and P (D) levels were tested in three groups, and AMH mRNA expression in the ovarian tissues (E) was also tested. Ten mice per group were examined. All data are shown as the mean ± s.d. Statistical significance: *P < 0.05 and **P < 0.01 vs the control group, ^#^ *P* < 0.05 and ^##^ *P* < 0.01 vs the d-gal group.

The d-gal treatment group had significantly increased serum FSH and LH levels (*P* < 0.05 and *P* < 0.01) and significantly decreased E2 and P levels (*P* < 0.01) compared to those in the control (Fig. 1A, B, C and D). Due to insufficient sensitivity and stability, an enzyme immunoassay (ELISA) was not used to test the serum anti-Müllerian hormone (AMH) level. Instead, *Amh* mRNA expression in the ovarian tissue was tested using q-PCR. Interestingly, *Amh* mRNA expression was significantly decreased in the d-gal treatment group compared with that in the control group (Fig. 1E). In the POF model induced by d-gal, curcumin treatment significantly decreased the serum FSH and LH levels (*P* < 0.05, Fig. 1A and B) and increased the serum E2 and *P* levels (*P* < 0.01, Fig. 1C and D) and the ovarian *Amh* expression level (*P* < 0.05, Fig. 1E).

### Protective effect of curcumin on follicular development in the POF model

**Figure 2:**
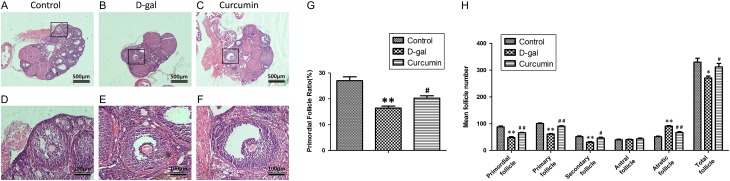
Effect of curcumin on follicular development in the POF model. Follicles were observed after H&E staining (A, B, C, D, E and F). The follicle classification was based on the characteristics proposed by Hirshfield and Midgley. (G) Primordial follicle ratio counting was performed in the three groups. Ten mice per group were examined. The primordial follicle ratio refers to the percentage of the primordial follicle number to the total follicle number. (H) The numbers of those follicles at different developmental stages of maturation were summarized. *P < 0.05 and **P < 0.01 vs the control group, ^#^ *P* < 0.05 and ^##^ *P* < 0.01 vs the d-gal group.

Follicle counting was performed after H&E staining (Fig. 2A, B, C, D, E and F). The follicle classification was based on the characteristics proposed by Hirshfield & Midgley (1978). Counting of the primordial follicles showed that d-gal treatment reduced the proportion of primordial follicles compared to that in the control group (*P* < 0.01, Fig. 2G and H). Significantly more follicles were counted at the different developmental stages of maturation in the curcumin treatment group than in the d-gal model group (*P* < 0.05 or *P* < 0.01, Fig. 2H).

### Effects of d-gal and curcumin on oxidative stress

**Figure 3:**
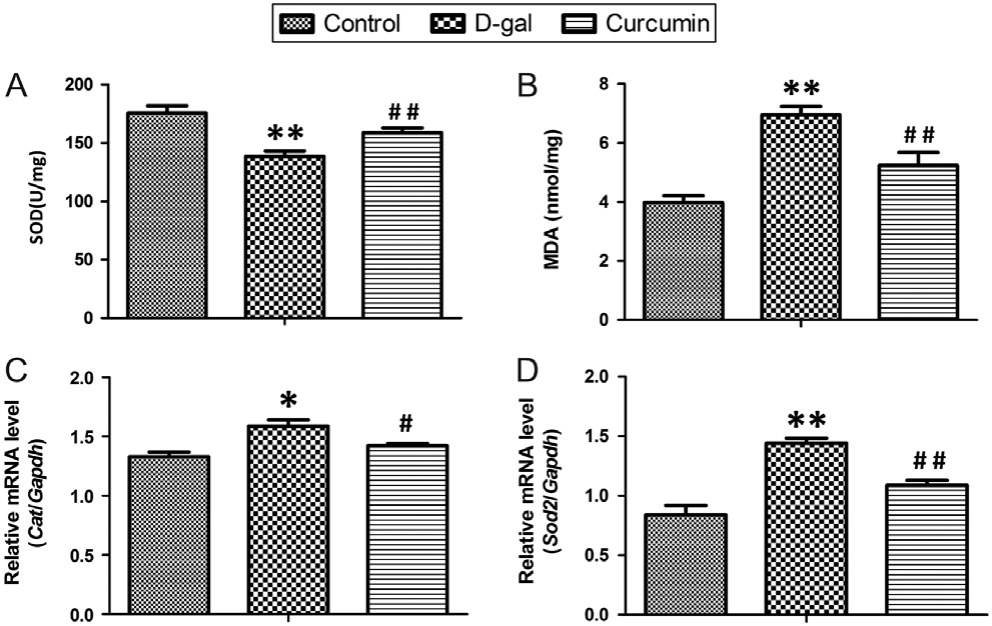
Effects of d-gal and curcumin on oxidative stress. The total SOD enzyme activity (A) and the MDA level (B) were measured in the ovarian tissues. *Cat* (C) and *Sod2* (D) mRNA expression was tested using Q-PCR. Ten mice per group were examined. All data are shown as the mean ± s.d. Statistical significance: *P < 0.05 and **P < 0.01 vs the control group, ^#^ *P* < 0.05 and ^##^ *P* < 0.01 vs the d-gal group.

The total SOD enzyme activity was significantly lower in the ovarian tissues from the d-gal treatment group than in the tissues from the control group (*P* < 0.01, Fig. 3A), and the MDA level was significantly higher in the treated group than in the control group (*P* < 0.01, Fig. 3B). Curcumin treatment resulted in markedly higher SOD levels and lower MDA levels than the levels in the d-gal group (all *P* < 0.01, Fig. 3A and B). *Cat* and *Sod2* mRNA expression was tested using Q-PCR (Fig. 3C and D). Corresponding with the changes in the SOD activity and MDA level, *Cat* expression was significantly increased in the d-gal group compared to that in the control group (*P* < 0.01), and curcumin treatment partially but significantly rescued the effect of d-gal (*P* < 0.05, Fig. 3C). However, the change in *Sod2* expression was seemingly abnormal. The *Sod2* mRNA expression level was significantly increased in the d-gal model (*P* < 0.01, Fig. 3D), whereas curcumin treatment partially and significantly rescued the effect of d-gal (*P* < 0.01).

### Protective effect of curcumin on d-gal-induced ovarian cell apoptosis

**Figure 4:**
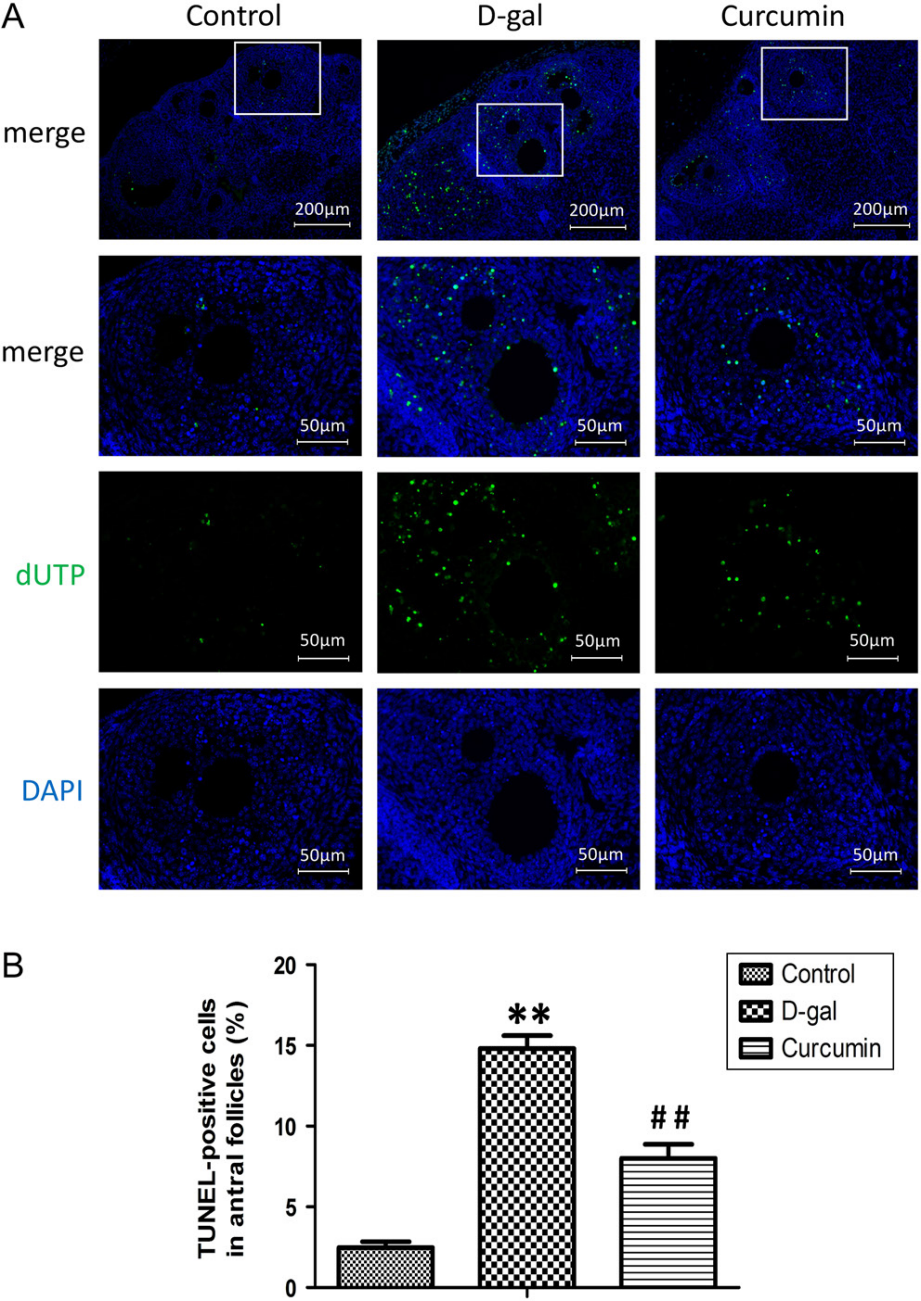
Effect of curcumin on d-gal-induced apoptosis of ovarian cells. Apoptosis was analyzed using *in situ* TUNEL fluorescence staining (A). In the TUNEL assay, the nuclei of TUNEL-positive (apoptotic) cells stained green. Five random fields per section (five sections per tissue from a mouse) were examined in each experiment. The TUNEL-positive granulosa cells and total granulosa cells in the antral follicles were counted. The number of TUNEL-positive granulosa cells (%) was compared among the three groups (*n* = 8) (B). Statistical significance: *P < 0.01 and **P < 0.01 vs the control group; ^#^ *P* < 0.01 and ^##^ *P* < 0.01 vs the d-gal group.

In the TUNEL assay, the nuclei of the TUNEL-positive (apoptotic) cells stained green (Fig. 4A). The number of apoptotic granulosa cells in the antral follicles was assessed in the three groups. More TUNEL-positive cells were detected in the d-gal group than in the control group (*P* < 0.01, Fig. 4B). Curcumin significantly decreased the number of TUNEL-positive cells compared with the number in the d-gal model group, suggesting a protective effect on d-gal-induced ovarian cell apoptosis (*P* < 0.01).

### Effects of curcumin on 4-HNE, NTY, 8-OHdG and p16 protein expression

**Figure 5:**
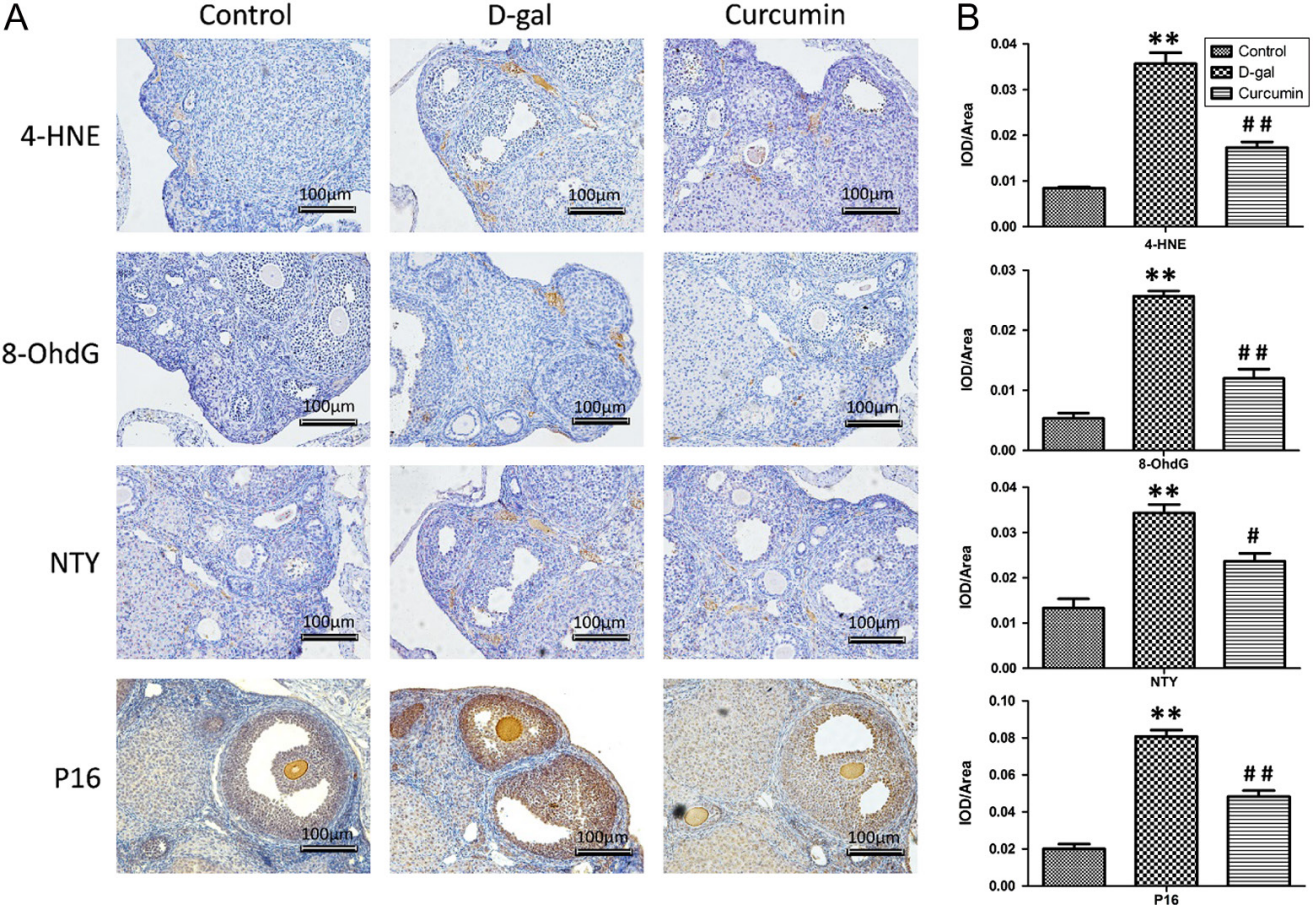
Effects of curcumin on 4-HNE, NTY, 8-OHdG and p16 protein expression. The cellular locations of these proteins were observed using immunohistochemistry (A), and 4-HNE, NTY, 8-OHdG and p16 expression was quantitatively analyzed (*n* = 8) (B). Data are shown as the mean ± s.d. Statistical significance: *P < 0.01 and **P < 0.01 vs the control group; ^#^ *P* < 0.05 and ^##^ *P* < 0.01 vs the d-gal group.

The cellular locations of the 4-HNE, NTY, 8-OHdG and p16 proteins were examined using immunohistochemistry (Fig. 5). The 4-HNE, NTY and 8-OHdG proteins were mainly located in the ovarian interstitial cells. The 4-HNE, 8-OhdG and NTY protein expression levels were significantly higher in the d-gal group than in the control group (*P* < 0.01, Fig. 5B), whereas the expression levels were partially and significantly decreased in the curcumin treatment group (*P* < 0.01 and *P* < 0.05, respectively).

Interestingly, the senescence-associated p16 protein was mainly located in follicular granulosa cells and oocytes (Fig. 5A), and relatively lower p16 expression was observed in the ovarian interstitial cells. Corresponding to the 4-HNE, 8-OhdG and NTY protein expression levels, d-gal treatment induced a significant increase in p16 protein expression, whereas curcumin treatment rescued the effect of d-gal treatment (*P* < 0.01, Fig. 5B).

### Effect of curcumin on related pathways in the ovary

**Figure 6:**
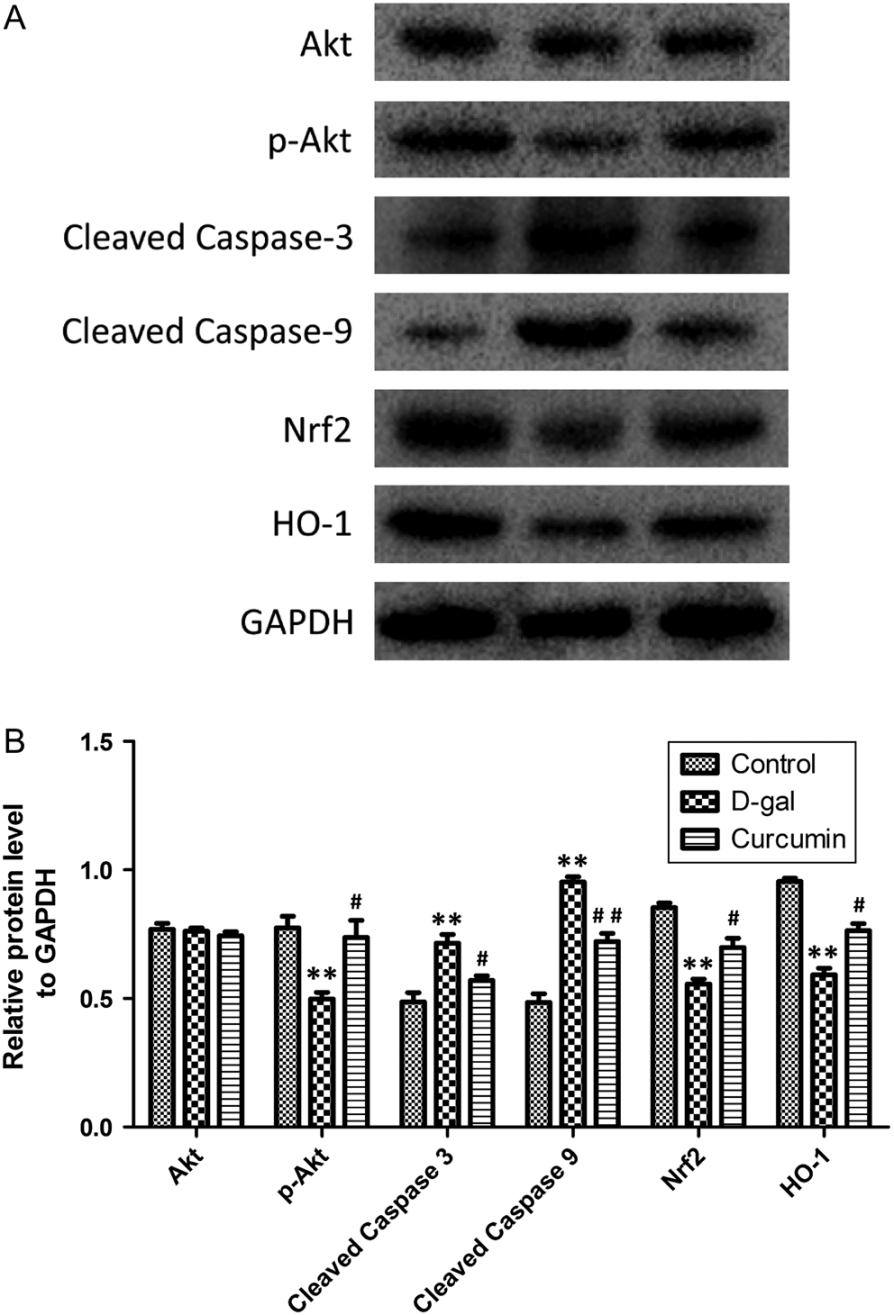
Effect of curcumin on the expression of apoptosis- and oxidative stress-associated factors in ovarian tissues. The Akt, p-Akt, cleaved caspase-3, cleaved caspase-9, Nrf2 and HO-1 protein expression levels in ovarian tissues were observed using Western blotting (A). The Akt, p-Akt, Nrf2 and HO-1 protein expression levels were quantitatively analyzed (*n* = 8) (B). Data are shown as the mean ± s.d. Statistical significance: *P < 0.01 and **P < 0.01 vs the control group; ^#^ *P* < 0.05 and ^##^ *P* < 0.01 vs the d-gal group.

We investigated the potential mechanisms involved in the effects of curcumin on d-gal-induced ovarian aging. The expression levels of apoptosis- and oxidative stress signal pathway-associated markers were assessed by Western blotting (Fig. 6). Total Akt protein expression was the same in each group, whereas the p-Akt level was markedly lower in the d-gal group than in the control group (*P* < 0.01) and was significantly higher in the curcumin group than in the d-gal group (*P* < 0.01). The cleaved caspase-3 and cleaved caspase-9 levels were significantly lower in the curcumin group than in the d-gal group (*P* < 0.01); moreover, the Nrf2 and HO-1 protein expression levels were significantly lower in the d-gal group than in the control group (*P* < 0.01), whereas the Nrf2 and HO-1 expression levels were significantly higher in the curcumin group than in the d-gal group (*P* < 0.05 and *P* < 0.01, respectively).

## Discussion

In the present study, a mouse POF model was successfully induced by d-galactose. d-galactose treatment resulted in increased ROS and AGEs, increased granulosa cell apoptosis and damaged follicular development. Curcumin partially rescued the effects of d-gal in the mouse POF model via a mechanism involving the Nrf2/HO-1 and PI3K/Akt signaling pathways.

Many studies have shown that d-gal directly induces oxidative stress* in vivo* and that galactose toxicity attenuates FSH bioactivity and inhibits E2 production from granulose cells (Banerjee *et al*. 2012). We found that the serum FSH and LH levels were significantly increased and the E2 and P levels were decreased in mice treated with d-gal. The number of atretic follicles was increased by the d-gal treatment; conversely, the decreased number of primordial, primary and secondary follicles resulted in a decreased number of total follicles, which was in line with a previous report (Bandyopadhyay *et al*. 2003). AMH is a very important early marker of ovarian aging (Visser *et al*. 2006, Soto *et al*. 2009, Tolikas *et al*. 2011) and reflects the size of the ovarian follicle pool (Feyereisen *et al*. 2006). Lower *Amh* expression has been detected long before normal menopause (Sanders *et al*. 2009, Seifer *et al*. 2011) and during the normal course of aging in mice (Kevenaar *et al*. 2006). We also found that ovarian *Amh* expression was significantly decreased by d-gal. Thus, the use of d-gal successfully induced the mouse POF model in the present study.

Curcumin is an antioxidant (Augustyniak *et al*. 2010), anti-inflammatory (Ueki *et al*. 2013), anti-apoptotic (Geng *et al*. 2017) and antibacterial (Mun *et al*. 2013) substance. Curcumin was demonstrated to exert a stimulatory effect on ovarian function, because curcumin promoted folliculogenesis and reduced apoptosis in murine ovarian cells (Voznesens’ka *et al*. 2010, Aktas *et al*. 2012). This study was designed to investigate the protective effects of curcumin on ovarian aging in a d-gal-induced mouse model of POF. We found that curcumin decreased the FSH and LH levels and increased the E2 and *P* levels in the mouse POF model. Interestingly, ovarian AMH expression was significantly increased by curcumin, whereas the total number of follicles increased with the increased numbers of primordial, primary and secondary follicles. Curcumin partially and significantly rescued the effects of d-gal in the POF model, suggesting that curcumin might regulate the reproductive endocrine function and promote follicular development or the maintenance of primordial follicles.

We found that the total SOD enzyme activity was decreased and the MDA level was increased in the d-galactose-induced POF model, whereas *Cat* and *Sod2* expression was significantly increased. Superoxide dismutase (SOD) is a type of enzyme that alternatively catalyzes dismutation of the superoxide (O^2−^) radical into either ordinary molecular oxygen (O_2_) or hydrogen peroxide (H_2_O_2_). The increased ROS production induced by d-gal is related to the inhibition of SOD activity. Three forms of superoxide dismutase (SOD1, SOD2 and SOD3) are expressed in all mammals. SOD2, which is the mitochondrial SOD and is also known as manganese-dependent superoxide dismutase (MnSOD), is a member of the iron/manganese superoxide dismutase family (Scandalios 2005). SOD2 and catalase are the two most important antioxidant enzymes in the body, and their expression levels are increased following oxidative stress (Scandalios 2005, Zhang *et al*. 2014). d-galactose, which is a reducing sugar that reacts readily with the free amines of amino acids in proteins and peptides to form AGEs (Bucala & Cerami 1992, Vlassara *et al*. 1994), produces acetaldehyde and hydrogen peroxide under the action of galactose oxidase and thus increases ROS, resulting in the aging of cells (Ho *et al*. 2003). Several studies have shown that oxidative stress can reduce the number of follicles and oocytes (Tarín 1995, 1996). Miyamoto *et al.* (2010) reported that oxidative stress caused a significant decrease in the number of follicles and the ovulated oocytes during repeated ovulation. We found that curcumin increased the total SOD activity and decreased MDA. Curcumin exerts a protective effect by inhibiting oxidative stress in a d-gal-induced model of ovarian aging, and its effects may be mediated via the suppression of ROS and superoxide anion free radicals (Suckow & Suckow 2006, Sikora *et al*. 2010).

The results showed that 4-HNE, NTY and 8-OHdG expression was mainly located in ovarian interstitial cells, and curcumin decreased the expression of those proteins. The d-gal-induced effects on ROS, AGEs and other factors on cellular aging should be extensive (Song *et al*. 1999). Factors such as 8-OhdG, 4-HNE and NTY, which are mainly located in somatic cells, are involved in the aging process related to ROS and AGEs. Interestingly, the p16 protein was highly expressed in ovarian follicles, including oocytes, granular cells and cumulus cells, and expressed at low levels in interstitial cells. The p16 protein negatively regulates cell proliferation and division and promotes apoptosis and senescence; thus, it affects the cell cycle and G1-S conversion by competitively suppressing the combination between CDK4/6 and cyclin D within G1 phase. We found that p16 expression in follicles was significantly increased in the d-gal-induced model and that curcumin significantly decreased d-gal-induced p16 expression. The p16 expression level was positively related to the follicular number and negatively related to the E_2_ and P levels. p16 expression was low in young animal tissues and subsequently increased with age (Kim *et al*. 2002, Attema *et al*. 2009). Excessive p16 expression can induce premature aging in cells (Attema *et al*. 2009). Baker and coworkers found that the aging cells reactivated and aging-associated phenotypes were reduced by silencing p16 protein expression (Baker *et al.* 2011). However, how p16 participates in ovarian aging through oxidative stress is unclear (Kim *et al*. 2002, Attema *et al*. 2009). We visualized an important effect of p16 on oxidative stress in ovarian follicles and found that curcumin also exerted a protective effect against ovarian aging partially through the downregulation of p16.

In the present study, we found that curcumin partially rescued the effects of d-gal via multiple mechanisms, including the inhibition of oxidative stress and granular cell apoptosis, as described in other reports (Suckow & Suckow 2006, Sikora *et al*. 2010). Our results also showed that curcumin significantly increased p-Akt, HO-1 and Nrf2 expression in ovarian tissues and decreased the expression of the apoptosis-related proteins cleaved caspase-3 and -9. PI3K/AKT signaling is widely accepted as a key pathway that regulates cell survival (Atif *et al*. 2015). Upregulation of phosphorylated PI3K and AKT can inhibit both the release of Bad and Bax and the activation of downstream pro-apoptotic proteins, such as caspases, resulting in the suppression of apoptosis (Rong & Distelhorst 2008, Atif *et al*. 2015). Nrf2 can bind to antioxidant response elements (AREs) in the promoter regions of Nrf2 target genes, which remove ROS via sequential enzymatic reactions, such as HO-1 (Gozzelino *et al*. 2010). The induction of HO-1 is an adaptive response to oxidative stress (Paine *et al*. 2010). Curcumin has also been reported to activate Nrf2 to upregulate enzymes involved in antioxidant defenses, including SOD and HO-1 (Balogun *et al*. 2003, Zingg *et al*. 2013). Our results indicated that curcumin attenuated d-gal-induced oxidative stress injury in the ovary partly by activating the PI3K/Akt and Nrf2/HO-1 pathways.

In conclusion, curcumin effectively inhibited d-gal-induced oxidative stress, apoptosis and ovarian injury via multiple mechanisms, including the Nrf2/HO-1 and PI3K/Akt signaling pathways. These results suggest that curcumin as an antioxidant is a potential protective agent against POF.

## Supplementary Material

Supporting Table 1

## Declaration of interest

The authors declare that there is no conflict of interest that could be perceived as prejudicing the impartiality of the reported research.

## Funding

This work was supported by the the National Key Research and Development Program of China (2017YFC1001602), Science and Technology Development Key Projects Fund of Nanjing Medical University (2016NJMUZD020), the Nature and Science Foundation of China (81270746), the Jiangsu Province Special Program of Medical Science (BL2012009 and ZX201110) and a project funded by PAPD of the Priority Academic Program Development of Jiangsu High Education Institutions (JX10231802).
