# Supplementary material for: Curcumin exerts a protective effect against premature ovarian failure in mice
Source: J Mol Endocrinol. 2018 Feb 7;60(3):261–71. doi: 10.1530/JME-17-0214 (PMC5863768; doi:10.1530/JME-17-0214)
Supplement: Supporting Table 1 [file jme-60-261-t001.pdf]

Supplementary information for:

Curcumin exerts a Protective Effect against Premature Ovarian Failure in Mice

Zhengjie Yan[a,b], Youjin Dai[c], Heling Fu[c], Yuan Zheng[c], Dan Bao[c], Yuan Yin[c], Qin Chen[c], Xiaowei Nei[d], Qingting Hao[c], Yugui Cui[b*], Daorong Hou[c*]

[a]College of Animal Science and Technology, Yangzhou University, 12 Wenhui East Road, Yangzhou, 225009, People's Republic of China

[b]State Key Laboratory of Reproductive Medicine, Center of Clinical Reproductive Medicine, The First Affiliated Hospital of Nanjing Medical University, 300 Guangzhou Road, Nanjing, 210029, People's Republic of China

[c]Animal Core Facility of Nanjing Medical University, Nanjing Medical University, 101 Longmian Avenue, Nanjing, 211166, People's Republic of China

[d]Department of Reproductive Medicine, Affiliated Hospital of Nanjing University of Traditional Chinese Medicine, 155 Hanzhong Road, Nanjing 210029, China.

*Correspondence and requests for materials should be addressed to Dr. Daorong Hou (houdaorong@njmu.edu.cn) or Prof. Yugui Cui (cuiygnj@njmu.edu.cn).

Supplementary Table 1. List of primers and condition for Q-PCR.

Genes	Primer sequence (5’-3’)	Accession Number	Products size（bp）	Annealing Temperature (℃)
Amh	5'-TCCTACATCTGGCTGAAGTGATATG-3'	NM_00744	166	58
	5'-CAGGTGGAGGCTCTTGGAACT-3'	5.2		
Cat	5'-GGTGCGGACATTCTACACAAAG-3'	NM_00980	162	52
	5'-TGTTCTCACACAGGCGTTTCC-3'	4.2		
Sod2	5'-GTGAACAATCTCAACGCCA-3'	NM_01367	189	51
	5'-GATAGCCTCCAGCAACTCT-3'	1.3		
Gapdh	5’-CTATTGGCAACGAGCGGTTCC-3’	DQ403054.	152	55
	CAGCACTGTGTTGGCATAGAGG	1		
